# Therapeutic Effects of Probiotic Minas Frescal Cheese on the Attenuation of Ulcerative Colitis in a Murine Model

**DOI:** 10.3389/fmicb.2021.623920

**Published:** 2021-03-02

**Authors:** Bárbara F. Cordeiro, Juliana L. Alves, Giovanna A. Belo, Emiliano R. Oliveira, Marina P. Braga, Sara H. da Silva, Luisa Lemos, Jonas T. Guimarães, Ramon Silva, Ramon S. Rocha, Gwénaël Jan, Yves Le Loir, Marcia Cristina Silva, Mônica Q. Freitas, Erick A. Esmerino, Alfonso Gala-García, Enio Ferreira, Ana Maria C. Faria, Adriano G. Cruz, Vasco Azevedo, Fillipe L. R. do Carmo

**Affiliations:** ^1^Instituto de Ciências Biológicas, Universidade Federal de Minas Gerais (UFMG), Belo Horizonte, Brazil; ^2^Department of Infectious Diseases, Harvard Medical School, Massachusetts General Hospital, Boston, MA, United States; ^3^Faculdade de Medicina Veterinária, Universidade Federal Fluminense (UFF), Niterói, Brazil; ^4^Departamento de Alimentos, Instituto Federal de Educação, Ciência e Tecnologia do Rio de Janeiro (IFRJ), Rio de Janeiro, Brazil; ^5^INRAE, STLO, Institut Agro, Agrocampus Ouest, Rennes, France

**Keywords:** functional food, probiotic, colitis, cheese, *Lactococcus lactis*, inflammatory bowel disease

## Abstract

Inflammatory bowel diseases (IBDs) constitute disturbances of gastrointestinal tract that cause irreversible changes in the structure and function of tissues. Ulcerative colitis (UC), the most frequent IBD in the population, is characterized by prominent inflammation of the human colon. Functional foods containing probiotic bacteria have been studied as adjuvants to the treatment or prevention of IBDs. The selected probiotic strain *Lactococcus lactis* NCDO 2118 (*L. lactis* NCDO 2118) exhibits immunomodulatory effects, with promising results in UC mouse model induced by dextran sodium sulfate (DSS). Additionally, cheese is a dairy food that presents high nutritional value, besides being a good delivery system that can be used to improve survival and enhance the therapeutic effects of probiotic bacteria in the host. Therefore, this work investigated the probiotic therapeutic effects of an experimental Minas Frescal cheese containing *L. lactis* NCDO 2118 in DSS-induced colitis in mice. During colitis induction, mice that consumed the probiotic cheese exhibited reduced in the severity of colitis, with attenuated weight loss, lower disease activity index, limited shortening of the colon length, and reduced histopathological score. Moreover, probiotic cheese administration increased gene expression of tight junctions’ proteins *zo-1*, *zo-2*, *ocln*, and *cln-1* in the colon and increase IL-10 release in the spleen and lymph nodes. In this way, this work demonstrates that consumption of probiotic Minas Frescal cheese, containing *L. lactis* NCDO 2118, prevents the inflammatory process during DSS-induced colitis in mice, opening perspectives for the development of new probiotic functional foods for personalized nutrition in the context of IBD.

## Introduction

Functional food products are defined as “natural or processed foods containing known or unknown biologically active compounds which provide a clinically proven and documented health benefit for the prevention, control or treatment of chronic diseases when in used in defined, effective and non-toxic amounts” (Rolim et al., 2020). Among them, functional foods containing probiotic bacteria have been proposed for being safe for consumption and have the ability to modulate the responses in the host by cellular components or metabolites produced (Rabah et al., 2017). In this context, there is a wide demand for new functional foods, in particular, foods enriched by the addition of probiotics, driving the food’s industry to develop new products proven effective for health (Sperry et al., 2018).

Cheese is one of the most consumed dairy food and comprises, from the nutritional view, a source with good nutritional value, given high contents of protein, minerals, and vitamins (Matera et al., 2018). Moreover, cheese, especially soft cheese, is an excellent delivery system to introduce probiotics into the gastrointestinal tract (GIT), due to it is anaerobic conditions created by the protein-fat contents, which can form complex coacervates that microencapsulated the probiotic bacteria and also high pH and low acidity present in this kind of the cheese (Silva et al., 2018). These coacervates reduce the contact with a highly acidic gut environment and thereby promote probiotic bacteria survival (Castro et al., 2015). Noteworthy, the Minas Frescal cheese is one of the most consumed cheese in Brazil and constitutes one important activity of the dairy industries, due to the high yield and absence of maturation period, which allows a quick return on investment and, consequently, lowers costs to the consumer (Sperry et al., 2018; Rocha et al., 2020). These features show that Minas Frescal cheese is a good candidate for manufacturing a new probiotic function dairy food.

*Lactococcus lactis* strain is a Gram-positive lactic acid bacteria (LAB) that exhibit simple metabolism and rapid growth and, due to that, are widely used in food fermentation (Da Silva et al., 2019). More specifically, NCDO 2118, used in this work, is a strain of *L. lactis* subsp. *lactis* isolated from frozen peas (Oliveira et al., 2014) and was previously demonstrated anti-inflammatory and immunomodulatory activities in the treatment of diseases, especially, in inflammatory bowel diseases (IBDs; Luerce et al., 2014). Furthermore, the functional analysis of *L. lactis* NCDO 2118 genome reflected a physiological adaptation ability to environmental changes like industrial processes and transit through the human GIT (Da Silva et al., 2019). These characteristics make *L. lactis* NCDO 2118 an excellent candidate to be introduced in probiotic functional foods.

IBDs, which include ulcerative colitis (UC) and Crohn’s disease (CD), are marked by periods of remission and relapse of an inflammation condition in the GIT and have a high prevalence in westernized countries, reaching about 0.5% of these populations (Silva et al., 2019). The etiology of IBD still not well understood, but scientific evidence suggests that that the genetic susceptibility, associated with intestinal microbiota alterations, causing an exacerbated immune response in the host is involved in IBD pathogenesis (Zhang and Li, 2014). UC is the most frequent condition of IBD in the population, affecting the large intestine, also called the colon. UC causes small irritation and ulcers in the colon, pain, diarrhea often with blood in the stool, and weight loss (Cordeiro et al., 2019). Nowadays, studies have shown that consumption of probiotic bacteria has therapeutic effects on UC, which is demonstrated to decrease the colon inflammation in a mouse model as well as in UC patients (Mañé et al., 2009; Luerce et al., 2014; Santos Rocha et al., 2014; Berlec et al., 2017; Jakubczyk et al., 2020; Rabah et al., 2020). Thus, this study hypothesizes that Minas Frescal cheese, made using *L. lactis* NCDO 2118, has a therapeutic effect in dextran sodium sulfate (DSS)-induced colitis mouse model.

## Materials and Methods

### Cheese Processing

The cheese processing was performed in accordance with Grom et al. (2020). Fifty liters of raw milk with 3.2% w/w fat (Núcleo Avançado de Tecnologia de Alimentos) was pasteurized for 15 s at 72°C (Model pro110, Arpifrio, São Paulo, Brazil), cooled to 37°C, and equally divided into two portions of 25 l, each for processing of conventional and probiotic Minas Frescal cheese. Then, 0.2 g/l of calcium chloride (Labsynth, Sao Paulo, Brazil) and 3 g/l of coagulant powder (Halamix power, Chr. Hansen) were added into the milk and maintained in a double-jacketed tank for 40 min to coagulate. After, 0.1 g/l at probiotic culture *L. lactis* NCDO 2118 [7–8 log colony-forming unit (CFU)/g] was added to the probiotic cheese, while no addition of lactic bacteria was performed on conventional cheese. The curd was cut, the cheese whey was removed, and the grains were put in 250-g plastic molds. Dry salting was performed by direct addition of 0.8% w/v NaCl on the cheese surface. Cheeses were packed and stored at 5°C.

### Physicochemical Analyses of Conventional and Probiotic Cheese

The proximate composition (moisture, protein, and fat) was evaluated according to the methodology previously described (BRASIL, 2006). To determine the moisture content of cheeses, we oven-dried 5 g of a sample at 100–105°C, for 24 h. Protein quantification and fat levels were determined by the Kjeldahl and Gerber methods, respectively (BRASIL, 2006). All results were expressed as g/100 g.

The content analysis of calcium and sodium levels in both kinds of cheeses were performed by inductively coupled plasma (ICP) optical emission spectrometry (Spectro Analytical Instruments, Kleve, Germany) previously described by Felicio et al. (2016). Sodium and calcium standards were used to obtain the calibration curves. Ten grams of samples was hydrolyzed, for 16 h, using 2 ml of nitric-perchloric acid solution (2:1), at 120 ± 2°C. Samples were heated in a digestion block (Technal, São Paulo, Brazil) to 100 ± 2°C for 1 h and maintained for more than 2 h, at 170 ± 2°C. Then, after the samples reach room temperature, we added 2 ml of nitric-perchloric acid and heated them again for a further 4 h at 170 ± 2°C.

To obtain the pH levels of both cheese, we inserted a digital pH meter electrode (Micronal, B-375, Digimed, Piracicaba, São Paulo, Brazil) into the diluted cheese samples as previously described (Silva et al., 2017).

### Bioactivity

To measure the bioactive peptides in cheese samples, we evaluate the angiotensin I-converting enzyme inhibitor (ACEI), antioxidant activity assay [2,2-diphenyl-1-picrylhydrazyl (DPPH)], and α-amylase and α-glucosidase inhibition.

The ACEI in probiotic and conventional cheese was determined by spectrophotometric assay, according to Konrad et al. (2014). The ACEI was calculated as follows: ACE inhibitory activity (%) = [(B − A)/(B − C)] × 100, where A is the absorbance in the presence of ACE and ACE components, B is the absorbance with ACE and without the ACE component, and C is the absorbance without the ACE or ACE component.

The DPPH radical-scavenging method previously described was used to determine the antioxidant activity capacity of cheeses (Lee et al., 2016). For that, 200 μl of 10% cheese sample was mixed with 1 ml of 100 μmol/l of DPPH solution. Besides that, as a positive control, butylated hydroxytoluene at 1 mg/ml concentration was used. After 15 min, the absorbance was measured at 517 nm using a spectrophotometer. The DPPH was calculated as follows: DPPH radical-scavenging activity (%) = [1 − (sample absorbance at 517 nm/control absorbance at 517 nm)] × 100.

The measurement of α-glucosidase and α-amylase inhibitory activities was determined according to Grom et al. (2020). The α-glucosidase inhibitory activity was determined dissolving 100 μl of α-glucosidase (0.2 units/ml) in 100 μl of phosphate buffer (pH 6.8), mixed with 150 μl of water-soluble extracts, and incubated for 20 min at 37°C. Then, 100 μl of 2.5 mM of p-nitrophenyl α-d-glucopyranoside was added to start the reaction. After incubation at 37°C for 20 min, the reaction was stopped, and 80 μl of sodium carbonate solution (0.2 mol/l) was added. The absorbance of *p*-nitrophenol was read at 405 nm using CMax Plus microplate reader (Promega, São Paulo, Brazil).

The α-amylase inhibitory activity was measured, and 100 μl of human salivary α-amylase (20 units/ml) with 100 μl of water-soluble extracts was added and incubated at 37°C for 20 min. Then, 250 μl of starch solution (10 g/l) in phosphate buffer (pH 6.8) was added, and the solution was incubated at 37°C for 5 min. To stop the reaction, 250 μl of dinitrosalicylic reagent (1% 3,5-dinitrosalicylic acid and 12% sodium potassium tartrate in 0.4 M of NaOH) was added and heated at 100°C for 10 min. After that, the sample was cooled at room temperature using a cold-water bath, and then 2.000 μl of distilled water was added to the mixture. The absorbance was performed at 540 nm using a spectrophotometer. The percentage (%) of α-glucosidase and α-amylase inhibition was calculated as described by Grom et al. (2020).

### Evaluation of Therapeutic Effects of Minas Frescal Cheese Containing *L. lactis* NCDO 2118 in the Dextran Sodium Sulfate-Induced Colitis Model

#### Animals

Conventional female C57BL/6 mice of 8 weeks of age, obtained at Universidade Federal de Minas Gerais (UFMG, Belo Horizonte, Brazil), were used in this work. They were housed in plastic cages in a room with controlled temperature (18–23°C), light cycle of 14-h light/10-h dark, relative humidity (40–60%), and *ad libitum* access to food and water. All experimental procedures realized in this work were approved by the Ethics Committee on Animal Experimentation of the Universidade Federal de Minas Gerais (CEUA-UFMG, Brazil) by protocol no. 364/2018.

#### Experimental Design and Dextran Sodium Sulfate-Induced Colitis

Prior to intragastric gavage, cheese samples were daily prepared resuspending 250 mg of each cheese, separately in 250 ml of phosphate buffer (pH 7.4; 1:1), and homogenized for 2 min using an IKA T 10 Basic Ultra Turrax homogenizer. Bacterial viability in both cheese solutions was determined by CFU counts.

The mice were divided randomly into six main groups, each containing six animals per group (Table 1). Groups 1–3 represented the healthy control group (no DSS) that received drinking water from the same source and consisted of group 1 received only water content (Group Naive); group 2 received conventional Minas Frescal cheese (group conventional ch.); and group 3 was given probiotic Minas Frescal cheese containing *L. lactis* NCDO 2118 (group NCDO ch.). All mice from groups 4–6 (experimental groups) received DSS (36–50 kDa, MP Biomedicals, CAT 260110, LOT Q5756), as the only drinking source, prepared to a concentration of 1.7% in filtered drinking water and provided to the animals daily, for 7 days, according to acute colitis model previously described (Wirtz et al., 2017). Animals from group 4 received only drinking water with DSS (group DSS) and no treatment; mice from group 5 were treated with conventional cheese (group DSS + conventional ch.), and group 6 were treated with probiotic Minas Frescal cheese containing *L. lactis* NCDO 2118 (DSS + NCDO ch.). For this experimental procedure, all mice received 0.5 ml of the respective treatments, in a single daily dose, by intragastric gavage, concomitantly with DSS induction (for 7 days). Each animal received approximately 2.5 × 10^6^ CFU/g of probiotic bacteria, per day, according to the results obtained by previous studies (Cordeiro et al., 2019; Rabah et al., 2020) and the adequate amount of bacteria for effect on the colon (Minelli and Benini, 2008). Mice were euthanized on the seventh day. All *in vivo* experiments were done in biological triplicate.

**Table 1:** Experimental groups and the respective treatments.

Healthy control group (consumption of drinking water)		Inflamed groups [consumption of DSS (1.7%) in the drinking water]	
Group	Treatment	Group	Treatment
Naive	H_2_O	DSS	H_2_O
Conventional ch.	Conventional cheese	DSS + conventional ch.	Conventional cheese
NCDO ch.	Probiotic cheese containing *L. lactis* NCDO 2118	DSS + NCDO ch.	Probiotic cheese containing *L. lactis* NCDO 2118

All groups were gavaged daily, with 0.5 ml of the appropriate treatments, for 7 days.

DSS, dextran sodium sulfate.

#### Assessment of Colitis Disease

Mouse body weight was individually measured during all experimental days. Water and food consumption were also recorded daily. The disease activity index (DAI) was determined on the last experimental day, as described by Cooper et al. (1993). This score measurement three major colitis clinical signs: weight loss, levels of diarrhea, and presence of rectal bleeding.

To access the intestine and colon for future assays, a longitudinal abdominal incision was performed in all mice. The colon length of each mouse was individually measured (from the cecum to rectum), and the values obtained were used to indicate the mean of each experimental group, in cm. Then, the distal portion of each colon was collected and washed with phosphate-buffered saline (PBS) for making colonic segment rolls for histomorphological analysis. These rolls were immersed in formaldehyde solution (4%, v/v) for tissue fixation, and after that, they were embedded in paraffin. A section (4 μm) was placed on a glass slide and stained with hematoxylin and eosin (H&E; Marchal Bressenot et al., 2015). Then, the sections were photographed (20× magnification objective) using a digital camera (Spot Insight Color) coupled to an optical microscope (Olympus, BX-41, Japan). The histological inflammation score was determined by a pathologist. To measure the level of histological inflammation in the colon tissue, the score previously described was used (Wirtz et al., 2017). This score considered the following features: tissue damage (0: none; 1: isolated focal epithelial damage; 2: mucosal erosions and ulcerations; 3: extensive damage deep into the bowel wall) and lamina propria inflammatory cell infiltration (0: infrequent; 1: increased, some neutrophils; 2: submucosal presence of inflammatory cell clusters; 3: transmural cell infiltrations). The total score ranging from 0 (no changes) to 6 (widespread cellular infiltrations and extensive tissue damage) was obtained by the sum of these two sub-scores (tissue damage and lamina propria inflammatory cell infiltration). Furthermore, to stain mucus-producing goblet cells, other cuts of the paraffinized colon samples were produced and stained by the Periodic acid-Schiff (PAS; Prisciandaro et al., 2011). Ten random field images from each sample were made using the 40× objective, and then with the use of ImageJ software (version 1.8.0), the intact goblet cells were counted. The total number of goblet cells was expressed as the number of cells per high-power field (hpf; 40×, 108.2 μm^2^).

#### Measurement of Secretory Immunoglobulin A

Secretory immunoglobulin A (sIgA) was determined by linked immunosorbent assay (ELISA), according to Cordeiro et al. (2018). For that, 96-well plates (Nunc-Immuno Plates, MaxiSorp) were coated with anti-IgA antibodies (Southern Biotechnology, Birmingham, AL, United States) and incubated overnight. Plates were washed in salina-Tween (salina with 0.05% of Tween-20; SIGMA Chemical Co) and blocked with 200 μl of PBS-casein (0.05%) for 1 h at room temperature. Intestinal lavage contents were added, and the plate was serially diluted (1:100) and incubated at room temperature for 1 h. Plates were washed with salina-Tween, and then, biotin-conjugated anti-mouse IgA antibody (Southern Biotechnology; 1:10,000 in PBS-casein) was added. Plates were incubated for 1 h at 37°C, and then, biotinylated monoclonal antibody anti-IgA (BD Biosciences) was added and incubated for 1 h at room temperature. Subsequently, peroxidase-labeled streptavidin (Southern Biotechnology) was added. Plates were washed in salina-Tween and incubated again with 100 μl of *o*-phenylenediamine (OPD; Sigma, St. Louis, MO, United States) and H_2_O_2_ (0.04%) for 1 h at room temperature. For stop reaction, 20 μl/well of 2 N of H_2_SO_4_ was added. Reading was performed on Bio-Rad Model 450 Microplate Reader at 492-nm absorbance. The results of total sIgA were measured, according to the standard curve, in a concentration of sIgA (ng) per ml of intestinal fluid.

#### Measurement of the Activity of Myeloperoxidase

The levels of neutrophil infiltration in the colon tissue were assessed by measurement of myeloperoxidase (MPO) activity, as previously described by Porto et al. (2019). For MPO quantification, a piece of colon tissue (10 mg) was homogenized proportionally in 1.9 ml/100 mg of PBS and centrifuged at 12,000 *g* for 10 min. The supernatant was discarded, and the pellet formed was lysed and centrifuged again. The supernatant formed was discarded again, and the pellet was resuspended proportionally in 1.9 ml/100 mg of 0.5% hexadecyltrimethyl ammonium bromide (HTAB) diluted in PBS. Afterward, were subjected to a freeze-thaw cycle (3×) using liquid nitrogen and then centrifuged at 12,000 *g* at 4°C for 10 min. To realize the enzymatic assay, we added an equal amount of substrate (1.5 mM/l of OPD and 6.6 mM/L of H_2_O_2_ in 0.075 mM/L of Tris-HCl pH 8.0) to the supernatant. To stop the enzymatic reaction, 50 μl of 1 M of H_2_SO_4_ was added. The absorbance was read in a spectrophotometer (Spectramax M3, Molecular Devices, LLC, Sunnyvale, CA, United States) at 492 nm. The results were expressed as arbitrary units (AU/mg).

#### Gene Expression Analysis in the Colon

The quantitative gene expression in colon fragment was determined according to Do Carmo et al. (2019). For that, fragments of 1 cm of the colon were collected, and then, the total RNA was isolated using PureLink RNA Mini Kit (Thermo Fisher Scientific) according to the manufacturer’s protocol. Afterward, DNase I (Invitrogen, Waltham, MA, United States) was used to digest residual genomic DNA of samples, and then Turbo DNA-free Kit (Ambion, Austin, TX, United States) was used for DNA removal following the manufacturer’s protocol. RNA quality was checked by agarose gel and NanoDrop® ND-1000 (260/230 ratio). To obtains the sample cDNA, the High-Capacity cDNA Reverse Transcription kit (Applied Biosystems, Foster City, CA, United States) was used. Quantitative PCR (qPCR) was determined using iTaq Universal SYBR Green Supermix (Bio-Rad, Hercules, CA, United States) and gene-specific primers, according to Do Carmo et al. (2019), for zonula occludens 1 and 2 (*zo-1* and *zo-2*, respectively), occludin (*ocln*), claudin-1 (*cln-1*), mucin-2 (*MUC-2*), inducible nitric oxide synthase (*iNOS*), and cytokine genes for interleukin-10 (*IL-10*), *IL-17*, *IL-1β*, as well as housekeeping genes encoding β-actin (*actβ*) and GAPDH (*gapdh*). The amplification cycles were performed as follows: 95°C for 30 s and 40 cycles of 95°C for 15 s and 60°C for 30 s on ABI PRISM 7900HT Sequence Detection System (Applied Biosystems). Results were expressed as a fold change of expression levels, using the mean and standard deviations of target expression (2^−ΔΔCT^).

#### Cell Preparation for Culture and Flow Cytometry

Cell suspension preparation for cytokine analysis and flow cytometry measurements were performed according to Canesso et al. (2018). Firstly, as UC affects the distal portions of the intestine, especially, the colon (Mizoguchi et al., 2020), we extracted the cecal lymph node (which drains the cecum) and the colonic lymph node (which drains the colon) for cell culture (Vieira et al., 2012; Esterházy et al., 2019). As the colon lymph node is very small, we did a pool mixing the two lymph nodes to reach enough cells for cell labeling. After that, the organs were macerated with sterile complete RPMI medium [containing 10% fetal bovine serum (FBS)] using a glass tissue macerator. Them, the organs were centrifuged and resuspended in a complete RPMI medium. For the spleen, cell-culture preparation was necessary to lysate the red blood cells, adding 9 ml of distilled water for 5 s. To stop this lysis process, 1 ml of PBS (10×) was added. The spleen capsule was removed to facilitate the presence of only immune cells. These cells were centrifuged and isolated from medium and then were incubated at 1 × 10^6^ cells per well, for cytokine secretion analyses, and another 1 × 10^6^ cells were incubated with antibodies for flow cytometry.

#### Cytokine Quantification by ELISA

Cells isolated from spleen and lymph node culture were cultured in 96-well plate (1 × 10^6^ cells/well) in sterile supplemented RPMI 1640 and stimulated or not with 1 mg/ml of anti-CD3 and anti-CD28, according to Canesso et al. (2018). The cells were incubated in an atmosphere of 5% CO_2_ for 48 h at 37°C, for measurement of IL-10, IL-17, and IL-1β cytokines, by ELISA, according to the manufacturer’s instructions (R&D Systems).

#### Flow Cytometry Analyses

Isolated cells from the spleen and lymph nodes were washed with PBS and pre-incubated with purified rat anti-mouse CD16/CD32 (Fc Block, clone: 2.4G2, BD Biosciences Pharmingen) for 20 min at 4°C to block FcγRII/III receptors. For surface staining, cells were incubated at 4°C for 30 min with anti-CD45.2 (FITC; clone: 104, BD Biosciences Pharmingen) and anti-CD4 (Pacific Blue, clone: RM4-5, BD Biosciences Pharmingen) fluorochrome-conjugated monoclonal antibodies. For intracellular staining, cells were first permeabilized following the *eBioscience Foxp3 Kit*, according to the manufacturer’s instructions, and later incubated with anti-FoxP3 (APC) [Alexa Fluor® 647, clone R16-715 (RUO), BD Biosciences Pharmingen], anti-LAP (PerCP-eFluor 710, clone: TW7-16B4, eBioscience), and anti-RORγt (PE; clone: Q31-378, BD Biosciences Pharmingen) fluorochrome-conjugated monoclonal antibodies for 30 min at 4°C. Individual controls (singles) were made containing only one labeled antibody in each tube, and also tubes with fluorescence minus one (FMO) were used. The gating strategy and the FMO controls are based on forward and side scatters, selecting splenocytes as a function of cell size and granularity (*n* = 6). Flow cytometry analysis was performed on a FACSCanto (BD Biosciences, San Jose, CA). The frequency (%) of positive cells and the mean fluorescence intensity were analyzed with the aid of the FlowJo program, version 10.0 (Tree Star, Ashland, OR, United States).

#### Statistical Analyses

Data were analyzed using one-way ANOVA followed by Tukey post-test and performed in GraphPad Prism version 7.00 for Windows (GraphPad Software, San Diego, CA, United States). The experimental assays were performed in triplicate, and the results were expressed as mean ± standard deviation. Asterisks demonstrated in all figures represent the significant differences between the experimental groups and were indicated as follows: ^*^p < 0.05, ^**^p < 0.01, ^***^p < 0.001, ^****^p < 0.0001.

## Results

### Proximate Composition and Mineral Content of Conventional and Probiotic Minas Frescal Cheese

Proximate composition (moisture, protein, fat, and lactose), sodium, calcium contents, and pH values are presented in Table 2. Our results showed that the addition of *L. lactis* NCDO 2118 did not affect significantly (*p* > 0.05) the proximate composition and mineral content of Minas Frescal cheese, compared with conventional cheese. Overall, probiotic cheese presented 68.2 ± 1.55, 17.5 ± 1.34, and 14.7 ± 1.13 (g/100 g) of moisture, protein, and fat, respectively, while conventional cheese presented 67.2 ± 1.43, 16.2 ± 1.56, and 14.4 ± 1.11, respectively. Regarding lactose amount, conventional cheese shows 2.2 ± 0.3, while probiotic cheese presented 1.8 ± 0.1 g/100 g. Regarding Na and Ca values, probiotic Minas Frescal cheese exhibited 549 and 316 mg/kg, respectively; meanwhile, conventional cheese presented similar values, 542 mg/kg of sodium and 312 mg/kg of calcium. pH values also did not present differences in both kinds of cheese, retaining a range of 5.

**Table 2:** Proximate composition and mineral contents of conventional and probiotic Minas Frescal cheese.

	Conventional cheese	Probiotic cheese
Moisture	67.2 ± 1.43	68.2 ± 1.55
Proteins	16.2 ± 1.56	17.5 ± 1.34
Fat	14.4 ± 1.11	14.7 ± 1.13
Lactose	2.2 ± 0.3	1.8 ± 0.1
Na	542 mg/kg	549 mg/kg
Ca	312 mg/kg	316 mg/kg
pH	5.52 ± 0.34	5.42 ± 0.21

Values are expressed as mean ± standard deviation. Moisture, protein, fat, and lactose values are expressed in g/100 g cheese, while sodium and potassium values are expressed in mg/kg. pH is dimensionless. Cheese analysis performed in triplicate.

### *Lactococcus lactis* NCDO 2118 on Cheese Enhances the Production of Bioactive Compounds

Table 3 shows the evaluation of bioactive compounds produced by conventional and probiotic cheese. Our results demonstrated that the antioxidant potential (DPPH), ACE inhibitory activity (ACEI), α-amylase, and α-glucosidase on the probiotic cheese, containing *L. lactis* NCDO 2118, presented increased values and were significantly different (*p* < 0.05) compared with conventional cheese. Regarding DPPH inhibition, we observed that values ranged from 22.3 ± 0.3% (convention cheese) to 43.3 ± 0.65% (probiotic cheese). Furthermore, probiotic cheese presented 32.4 ± 1.13% of ACEI, while the conventional cheese presents only 13.3 ± 0.73%. Likewise, probiotic cheese presented the highest values of α-amylase and α-glucosidase (28.9 ± 0.99% and 16.7 ± 1.12%, respectively), while conventional cheese presented 19.2 ± 1.45% and 10.3 ± 0.91%, respectively.

**Table 3:** Bioactive compounds from conventional and probiotic cheese.

	Conventional cheese	Probiotic cheese
DPPH	22.3 ± 0.3b	43.3 ± 0.65a
ACEI	13.3 ± 0.73b	32.4 ± 1.13a
α-Amylase	19.2 ± 1.45b	28.9 ± 0.99a
α-Glucosidase	10.3 ± 0.91b	16.7 ± 1.12a

Values are expressed in mean ± standard deviation. a–b Different letters in the same row indicate a significant difference (*p* < 0.05). ACEI, DPPH, α-amylase, and α-glucosidase are expressed as a percentage of inhibition (%). Cheese analysis performed in triplicate.

ACEI, angiotensin I-converting enzyme inhibitor; DPPH, 2,2-diphenyl-1-picrylhydrazyl.

### Treatment of Probiotic Cheese Did Not Alter the Liquid and Food Consumption or Caloric Intake of Mice

**Figure 1:**
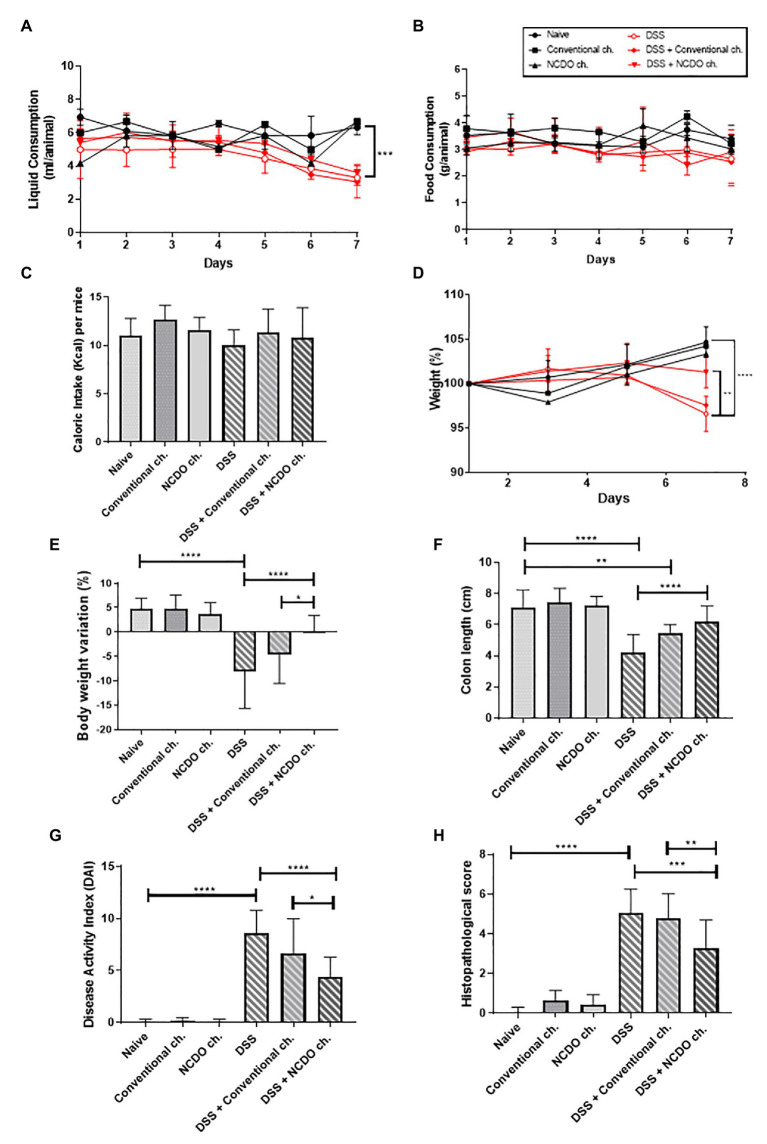
Impact of treatment with probiotic cheese on mice. **(A)** Liquid intake, **(B)** food consumption, and **(C)** caloric intake of mice across the different experimental groups. **(D)** Time course of mouse body weight monitoring during the seven experimental days. **(E)** Body weight loss observed on the seventh day of dextran sodium sulfate (DSS) colitis induction, and differences across the groups. **(F)** Changes in mouse colon length. **(G)** Disease activity index (DAI), a composite measure of weight loss, stool consistency, and presence of blood in stool. **(H)** Histopathological score obtained in mice. Values indicate the mean ± standard deviation. The data represent the mean ± SD (*n* = 6). Asterisks represent statistically significant differences, as follows: ^*^p < 0.05, ^**^p < 0.01, ^***^p < 0.001, ^****^p < 0.0001.

Figure 1 shows the liquid consumption (Figure 1A), the total food consumption (Figure 1B), and the caloric intake per mice (Figure 1C) during the experimental procedure. We observed a decrease in liquid intake on groups of mice that consumed water solution containing 1.7% of DSS over the experimental day, exhibiting the lowest consumption on the seventh day (3.3 ± 0.45 ml/animal). On the other hand, this consumption remains stable in groups receiving only drinking water (6.3 ± 0.441 ml/animal per day; *p* < 0.001). No differences were observed in liquid consumption in mice treated with conventional or probiotic cheese (*p* > 0.05) in health or unhealthy mice. Mice of all experimental groups consumed, on average, the same amount of food (3 g/animal per day), with no statistical difference between any experimental groups studied. Concerning caloric intake (Figure 1C), no difference between experimental groups has found by the intragastric gavage with conventional or probiotic cheese. Thus, both kinds of cheese did not alter the daily caloric content.

### Probiotic Minas Frescal Cheese Reduced the Weight Loss in Dextran Sodium Sulfate-Induced Ulcerative Colitis Mice

The consumption of probiotic cheese, containing *L. lactis* NCDO 2118 strain, was challenged in the DSS-induced colitis model. Mouse weight loss, monitored during the DSS administration, showed that the animals receiving DSS exhibited body weight loss starting from the third day, after DSS consumption (Figure 1D). Otherwise, mice from all healthy control groups presented a significant weight gain throughout the experiment days (*p* < 0.0001). Figure 1E shows that treatment with probiotic cheese has a protective effect on colitis-induced body weight loss. Mice from the DSS group that did not receive any treatment showed a marked weight loss (−8.08 ± 2.09%); however, mice treated with the probiotic cheese showed significant improvement in body weight (+0.23 ± 0.80%, *p* < 0.0001). Body weight variation was also statistically different (*p* < 0.05) between DSS + conventional cheese (−4.5 ± 5.9%) and DSS + NCDO cheese (+0.23 ± 0.80%).

### Probiotic Minas Frescal Cheese Alleviated Clinical and Macroscopic Signs of Colitis Disease

The shortening of colon length (Figure 1F) and the DAI (Figure 1G) were analyzed to verify major colitis macroscopic and clinical symptoms. Our results showed that the administration of DSS causes a pronounced shortening in the colon length (4.2 ± 1.12 cm) when compared with the naive group (7.0 ± 1.15 cm, *p* < 0.0001). However, the treatment with probiotic cheese, containing *L. lactis* NCDO 2118, prevents the shortening of the colon (6.2 ± 0.99 cm), being statistically different (*p* < 0.0001) when compared with the DSS group and results in a similar length to healthy animals (*p* > 0.05). Regarding DAI analyses, we observed that the administration of DSS was able to increase significantly (8.6 ± 2.1, *p* < 0.0001) the DAI score, compared with healthy groups (0.06 ± 0.2). Nevertheless, the intake of probiotic cheese was able to decrease significantly the DAI score (4.3 ± 1.8) compared with the DSS group (*p* < 0.0001) or with DSS + conventional cheese group (6.6 ± 3.3, *p* < 0.05).

### Colon Mucosal Damages Were Reduced in Mice Treated With Probiotic Minas Frescal Cheese

**Figure 2:**
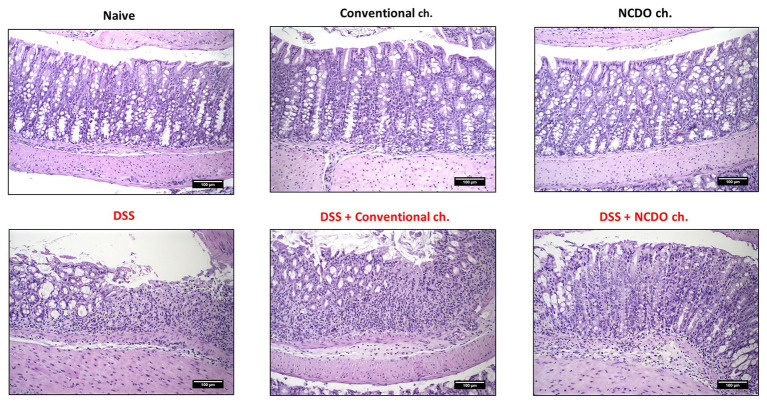
Effect of probiotic cheese administration on the dextran sodium sulfate (DSS)-induced histopathological damages. Representative H&E staining of colon mucosa sections. Image acquisition was done with a 20× magnification objective. Scale bar = 100 μm (*n* = 6).

Figures 1H, 2 reveal the impact of DSS administration and the effect of the probiotic treatment on the morphological structure of the mouse colon. Histopathological score (Figure 1H) and histological slide analysis (H&E staining, Figure 2) show that mice subjected to DSS consumption presented alterations in the morphological architecture of the colon, with extensive damage deep into the tissue, erosions and ulcerations in the colon of some mice, and increased inflammatory cell infiltration. However, consumption of probiotic cheese in DSS colitis mice was able to ameliorate these mucosal damages. Mice from healthy control groups showed a null histological score, while the DSS group presented a score on average of 5.0 ± 1.1 (*p* < 0.0001). Consumption of probiotic cheese, in turn, decreases the score to 3.2 ± 1.4, being statistically different to the DSS group (*p* < 0.001) and DSS + conventional cheese group (4.8 ± 1.2, *p* < 0.01).

### Treatment With Probiotic Minas Frescal Cheese Prevented Degeneration of Goblet Cells and Improved Secretory IgA Production

**Figure 3:**
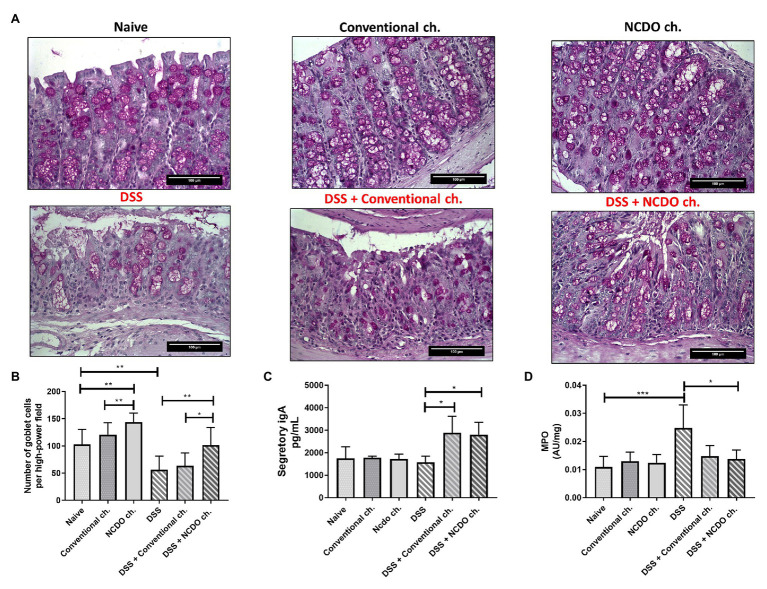
Effect of probiotic cheese administration on goblet cells, secretory immunoglobulin A (sIgA) levels, and the enzyme activity of myeloperoxidase (MPO). **(A)** Representative Periodic acid-Schiff (PAS) staining of colon mucosa sections. **(B)** Number of goblet cells, given by counting intact cells in 10 random field images on mouse colon. **(C)** Quantification of sIgA in the small intestine. **(D)** Levels of MPO activity that indirectly determines the neutrophil concentration in 10 mg of colon tissue. Values indicate the mean ± standard deviation (*n* = 6). Image acquisition was done with a 20× magnification objective. Scale bar = 100 μm (*n* = 6). Asterisks represent statistically significant differences: ^*^p < 0.05, ^**^p < 0.01, ^***^p < 0.001.

The administration of DSS provokes a substantial decrease in the number of goblet cells in the colon tissue (56.4 ± 25 goblet cell/hpf) when compared with the naive group (103 ± 27.5, *p* < 0.01, Figures 3A,B). Nonetheless, consumption of probiotic Minas Frescal cheese was still able to prevent this degeneration of goblet cells (101 ± 32.2 goblet cell/hpf), when compared with the DSS group (*p* < 0.01). Interestingly, the consumption of probiotic cheese provokes an improvement in the number of intact goblet cells, and also in the healthy control group (143.9 ± 16.3 goblet cell/hpf). Figure 3C shows levels of sIgA in the small intestine of mice. Our results showed that consumption of probiotic Minas Frescal cheese was able to increase the levels of sIgA (2,800.4 ng/ml) when compared with the naive group (1,750.3 ng/ml) and the DSS group (1,579.7 ng/ml).

### Probiotic Cheese Reduced the Inflammatory Cell Infiltration

In this work, we assessed the presence of colon neutrophil infiltrates by detecting its specific MPO enzymes (Figure 3D). Our results showed that mice in the DSS group had an inflammatory infiltrate with a very high level of neutrophils (0.0248, 0.008, *p* < 0.001) when compared with the naive group (0.0109 ± 0.003). However, when mice were treated with probiotic cheese, we found a significant reduction of these cells (0.0137, ± 0.003, *p* < 0.05), showing MPO levels very similar to those found in healthy animals (*p* > 0.05). Interestingly, we observed that conventional Minas Frescal cheese also presented reduced values of MPO (0.0148), being statistically different from those of the DSS (*p* < 0.05) group and similar to those of the DSS + NCDO group.

### Probiotic Minas Frescal Cheese Modulated Gene Expression in the Mice Colon

**Figure 4:**
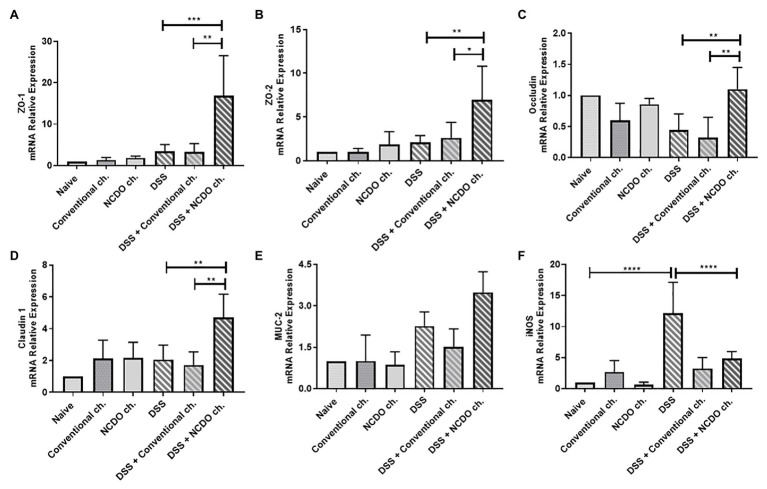
Impact of probiotic cheese on colonic expression markers of cell barrier and oxidative stress. Colonic mRNA expression levels of **(A)** *zo-1*, **(B)** *zo-2*, **(C)** *ocln*, **(D)** *cln-1*, **(E)** *MUC-2*, and **(F)** *iNOS* genes analyzed by qRT-PCR. The data represent the mean ± SD (*n* = 6). Asterisks represent statistically significant differences: ^*^p < 0.05, ^**^p < 0.01, ^***^p < 0.001, ^****^p < 0.0001.

**Figure 5:**
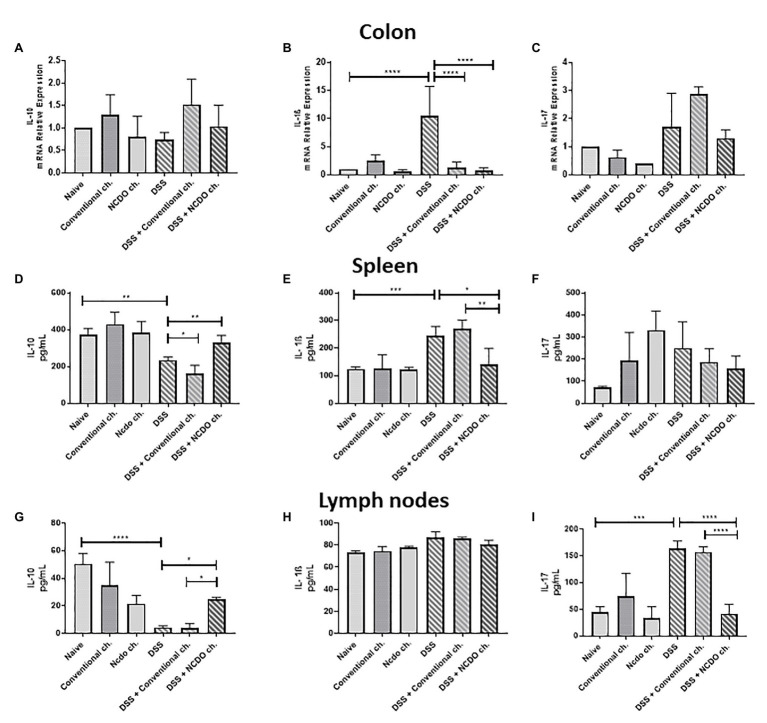
Colonic expression of cytokines genes and immunomodulatory effect of probiotic cheese in dextran sodium sulfate (DSS) colitis mice. Colonic mRNA expression levels of **(A)** *IL-10*, **(B)** *IL-1β*, and **(C)** *IL-17* genes analyzed by qRT-PCR. Enzyme-linked immunosorbent assay (ELISA) of **(D,G)** IL-10, **(E,H)** IL-1β, and **(F,I)** IL-17 cytokines in spleen and lymph node cell culture supernatant, respectively. The data represent the mean ± SD (*n* = 6). Asterisks represent statistically significant differences: ^*^p < 0.05, ^**^p < 0.01, ^***^p < 0.001, ^****^p < 0.0001.

In this work, we sought to evaluable the colonic mRNA expression levels of epithelial barrier genes (*zo-1*, *zo-2*, *ocln*, and *cln-1*), production of mucin gene (*MUC-2*), colonic oxidative stress (*iNOS*; Figure 4), and cytokine gene expression (*IL-10*, *IL-1β*, and *IL-17*; Figures 5A–C). Our results showed that the consumption of conventional or probiotic cheese in healthy control groups (naive, conventional ch., and NCDO ch. groups) was not able to alter the expression of the genes evaluated (*p* > 0.05). The intake of DSS in drinking water also did not alter the expression of *zo-1*, *zo-2*, *ocln*, *cln-1*, *MUC-2*, *IL-10*, and *IL-17* genes, when compared with the naive group. On the other hand, we observed an increase in the expression of *iNOS* and *IL-1β* genes, when compared with the DSS group and naive group (*p* < 0.0001). Interestingly, we observed that the consumption of probiotic cheese in unhealthy mice induced an increase in the expression of *zo-1*, *zo-2*, *ocln*, and *cln-1* genes of epithelial barrier, compared with the DSS and naive groups, while the expression of *iNOS* and *IL-1β* genes was decreased in animals treated with probiotic cheese. Also, we observed that *MUC-2* gene expression tended to increase in animals treated with probiotic cheese but was not statically different from DSS (*p* > 0.05).

### Probiotic Cheese Modulated Cytokine Production in Mice

To clarify the potential mechanisms by which probiotic cheese exerts its beneficial effects, we evaluated the cytokine profiles in the spleen and lymph nodes of mice (Figure 5). Our data showed that oral administration of probiotic cheese increased the levels of the anti-inflammatory cytokine IL-10 in the spleen (329.4 pg/ml, Figure 5D) and in the lymph nodes (24.9 pg/ml, Figure 5G), when compared with the DSS group (233.6 and 4.24 pg/ml, respectively) and DSS + conventional ch. (161.4 and 3.73 pg/ml, respectively). The intake of DSS led to increased cytokine IL-1β in the spleen (243.7 pg./ml, Figure 5E) and IL-17 in the lymph nodes (163.9 pg./ml, Figure 5I), when compared with the naive group (123.8 and 44.4 pg./ml). On the other hand, consumption of probiotic cheese in the DSS mice group was able to maintain IL-1β and IL-17 levels of production similar to healthy animal levels (140.9 and 41.53 pg/ml, respectively).

### Probiotic Bacteria, *L. lactis* NCDO 2118, Did Not Alter the Frequency of Tregs in the Spleen and Lymph Nodes

**Figure 6:**
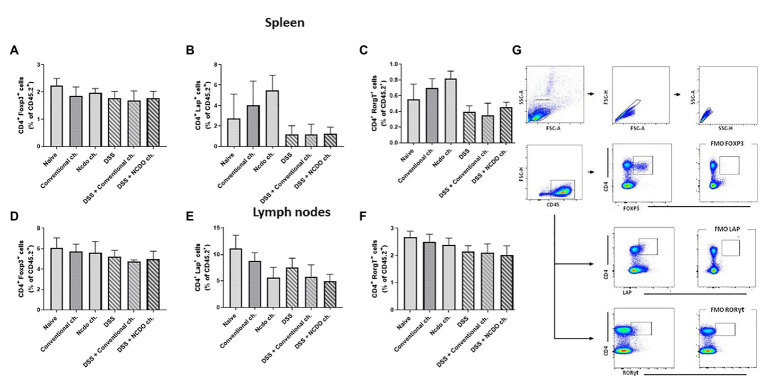
Evaluation of T lymphocyte production in spleen and lymph nodes after induced colitis. T cells were isolated from mouse spleen and lymph node, and the frequencies of CD4^+^Foxp3^+^ **(A,D)**, CD4^+^RORγt+ T cells **(B,E)**, and CD4^+^Lap+ T **(C,F)** cells as frequency (%) of CD4^+^ T cells were assessed by flow cytometry. **(G)** Plots with the gating strategy based on forward and side scatters, selecting splenocytes as a function of cell size and granularity (*n* = 6).

T-cell subpopulation (Treg CD4^+^Foxp3^+^, CD4^+^LAP^+^, and CD4^+^RorγT^+^) was evaluated in mice spleen and lymph nodes by using flow cytometry (Figure 6). The probiotic cheese, containing *L. lactis* NCDO 2118, did not change the percentage of Tregs on the spleen and lymph nodes in both healthy and inflamed mice. No statistical differences were found between the DSS and naive groups for all cells analyzed here.

## Discussion

The importance of diet in human health has been described by various scientific evidence; therefore, the development of new food products with health-giving additives and medical benefits is a pressing need (Domínguez Díaz et al., 2020). In this context, probiotic functional foods have been proposed, due to the proven therapeutic benefits of probiotic bacteria by the consumers (Carmo et al., 2017). In this work, we developed a new probiotic Minas Frescal cheese for the treatment of UC.

The addition of certain bacteria to cheese can contribute to altering glycolysis, proteolysis, and lipolysis processes that change the proximate composition and mineral contents of cheese and modify the organoleptic properties of the final product (Cárdenas et al., 2014). Thus, it was necessary to investigate whether the Minas Frescal cheese manufactured with *L. lactis* NCDO 2118 altered the centesimal composition and bioactive compounds of the cheese, as well as whether Minas Frescal cheese was a good matrix to maintain the viability of this probiotic strain. It is worth emphasizing that the beneficial effects of foods containing probiotics strains depend on the ability of these bacteria to survive to industrial process after passing through the GIT, which imposes unfavorable bacterial conditions and can affect probiotic potential (Cordeiro et al., 2019). In this sense, the regulatory agencies around the world recommended that for a probiotic product to be able to exercise its benefits, there must be a viable amount of probiotic bacteria of between 10^6^ and 10^7^ CFU/g (Castro et al., 2015). In this work, we observed that after manufacturing of Minas Frescal cheese, *L. lactis* NCDO 2118 presented 10^7^ CFU/g of viable cells counts, according to the recommendation, and this reinforces that Minas Frescal cheese is a good delivery system to maintain the viability of this probiotic bacteria through manufacturing processes. Previous studies showed that soft cheeses, like Minas Frescal cheese, are a good protective matrix for bacteria (Hosoya et al., 2012; Lollo et al., 2012, 2015; Sperry et al., 2018). In addition, our work demonstrated that *L. lactis* NCDO 2118 added on Minas Frescal cheese did not alter the proximal composition parameters evaluated (moisture, protein, fat, and lactose), as well the sodium and calcium contents, and pH values of the cheese. The probiotic cheese developed in this work still maintained the specifications recommended by the Brazilian legislation law established for moisture (>55%) and fat (25–44.9%) in Minas Frescal cheese dry matter (FDM; Matera et al., 2018).

Cheeses are recognized not only for their high nutritional value but also for the production of bioactive peptides, from casein hydrolyzed by proteases and peptidases (López-Expósito et al., 2017). Some of these peptides can resist gastrointestinal digestion, responsible for biological activities such as antihypertensive, antioxidant, and antidiabetic activities (Livney, 2010). However, the introduction of some probiotic bacteria, i.e., LAB, in this dairy product can increase the production of bioactive peptides (Smacchi and Gobbetti, 2000; Ayyash et al., 2018). Sperry et al. (2018) suggested that *Lactobacillus casei* 01 can generate high levels of antihypertensive (ACE-I) and antioxidant peptides (DPPH) in Minas Frescal cheese. In this sense, our probiotic Minas Frescal cheese, with *L. lactis* NCDO 2118, also induced an increased amount of antihypertensive (ACEI), antioxidant (DPPH), and antidiabetic activities (α-amylase and α-glucosidase) when compared with conventional Minas Frescal cheese. Interestingly, it is recognized that oxidative stress (OS) is one of the factors involved in the onset of IBD symptoms (Moura et al., 2015); therefore, we suggest that the increase in DPPH levels in probiotic Minas Frescal Cheese could help to ameliorate inflammation conditions in UC mice.

Considering the cheese properties presented, we decided to exploit the therapeutic effect of the consumption of the probiotic Minas Frescal cheese, containing *L. lactis* NCDO 2118, in the context of DSS-induced colitis in mice.

The UC disease symptoms include weight loss, tummy pain, recurring diarrhea with blood in the stool, and malaise (Zhang and Li, 2014). The inflammation reaches the mucosa and submucosa layers of the colon section, with the presence of edema, significant depletion of goblet cells, and changes in tissue architecture and ulcerations (Cordeiro et al., 2019). UC treatments are based on the control of the symptoms and administration of anti-inflammatories and antibiotics, immunosuppressive drugs, and surgeries in severe cases. However, none of these treatments are curative and instead provoke serious collateral effects in UC patients (Chibbar and Dieleman, 2015). In this context, functional probiotic foods have been suggested to be used alone or in combination with conventional drugs and act like adjuvant therapy to enhance remission in UC patients (Rabah et al., 2020). Regarding the mice weight loss triggered by DSS administration, we observed that the treatment with the probiotic Minas Frescal cheese, for seven experimental days, was able to prevent weight loss in mice. It is important to clarify that no differences in food consumption or caloric intake were observed in all groups analyzed, which suggested that this weight gain is linked to probiotic cheese administration. As in previous studies, consumption of probiotic bacteria in a dairy food was able to prevent weight loss in inflammatory disorder mouse models (Santos Rocha et al., 2014; Plé et al., 2016; Cordeiro et al., 2018).

In this work, we observed that unhealthy mice treated with probiotic Minas Frescal cheese exhibited attenuated clinical and macroscopic signs of colitis disease. This is mainly demonstrated by a decrease in DAI, hence, less diarrhea and rectal bleeding as well as the prevention of colon shortening triggered by DSS action. Similarly, Luerce et al. (2014) demonstrated that *L. lactis* NCDO 2118 improved the clinical signs of colitis by reducing the macroscopic inflammatory score of the disease, also seen by Rabah et. al (2020) who observed a reduction in the signs of UC induced by DSS by the consumption of a probiotic Emmental cheese.

Pathological assessment of UC is evidenced by extensive architectural damage of colon tissue, with erosions and ulcerations and depletion of the mucosal surface. Moreover, there was an increase in inflammatory cell infiltration in the *lamina propria*, i.e., neutrophil infiltrates, and also depletion of goblet cells (Jeengar et al., 2017). The activity of MPO is an indicator of this extent of neutrophil infiltrates in the mucosa (Ivanovska et al., 2017). Nevertheless, our results showed that the administration of probiotic Minas Frescal cheese protects the colon mucosa from DSS injury, marked by a decrease in the histological score and also a decrease in MPO levels. Besides that, we observed that probiotic cheese was able to decrease the expression of *iNOS* gene. This gene encodes the enzyme responsible for the generation of cytotoxic and immunoregulatory free radical NO, which is related to several inflammatory processes (Sakthivel and Guruvayoorappan, 2013). These results together demonstrated that there is less inflammation in the colon tissue of DSS mice treated with probiotic cheese.

Probiotic Minas Frescal cheese, with *L. lactis* NCDO 2118, was also able to preserve the number of intact goblet cells in the colon mucosa. These cells are responsible for producing the mucus that covers the intestinal mucosa. Considering that this mucus contains high levels of sIgA, we suggested that the increased sIgA levels, observed in the intestinal content of mice, were driven by the maintenance of the number of goblet cells due to the consumption of probiotic cheese. Precisely, mucus production by goblet cells and increased levels of sIgA were reported to be some of the mechanisms of probiotic action in the host (Rogier et al., 2014). Interestingly, dairy milk can significantly induce the host response to pathogens, enhance the integrity of the mucus layer (Tong et al., 2020), and increase secretory IgA in the small and large intestines (Schofield and Palm, 2018). In our previous works, we verified that dairy milk matrices, including cheese matrix, can increase IgA secretion (Cordeiro et al., 2018 Rabah et al., 2020), while the milk matrix shows an increase in the number of goblet cells (Cordeiro et al., 2018). Thus, this would explain the increases in the levels of sIgA and goblet cells found in the group treated with our probiotic cheese. On the other hand, to uncover the exact mechanisms, the expression of intestinal immune-related gene and sIgA levels needs to be better explored. Besides that, it is recognized that the presence of the mucus in the gut prevents the adhesion of microorganisms to the mucosa and their translocation into the lumen (Grondin et al., 2020). Moreover, the mucus is important for the lubrication and protection of the intestinal epithelium from toxic substances coming from the external environment, such as DSS (Abrantes et al., 2020). Thus, in mouse colon inflammation caused by DSS intake, it is common to observe a decrease in goblet cell number, but it can be restored by the consumption of probiotic bacteria (Rodrigues et al., 2018; Zhang et al., 2018; Abrantes et al., 2020). It is important to highlight that MUC-2 is the major glycoprotein constituent of intestinal mucus and is secreted primarily by goblet cells (Perez-Vilar, 2007). Interestingly, we also observed an increase in *MUC-2* gene expression in mice treated with probiotic cheese, corroborating with the observed increased production of the mucus in these mouse groups. As seen in a previous study, probiotic strain can stimulate *MUC-2* expression in intestinal goblet cells and mitigate acute colitis in a mouse model (Ma et al., 2020). Moreover, our findings showed that probiotic cheese administration also increased the gene expression of *zo-1*, *zo-2*, *ocln*, and *cln-1*. These genes are responsible for the expression of tight junction proteins that maintaining the epithelial barrier and control cellular permeability (Landy et al., 2016).

The host’s cytokine-mediated immune response plays a pivotal role during the development of acute colitis (Ko and Auyeung, 2014). Probiotic bacteria have a great ability to promote increased levels of anti-inflammatory cytokines and also to lead to a decrease in the production of pro-inflammatory cytokines (Carvalho et al., 2017). In IBD, IL-17 and IL-1β cytokines are related to the extensive lymphocyte, plasma cell, and macrophage infiltration into the tissue (Melgar et al., 2005; Shen and Durum, 2017). The decrease in the transcriptional colonic levels of IL-1β and the secretion of IL-1β and IL-17 (spleen and lymph node, respectively) by the consumption of probiotic cheese in the disease DSS group can be mediated by the action of IL-10 secretion (spleen and lymph node). IL-10 is the most important cytokine to control homeostasis in the intestinal mucosa (Sun et al., 2018), and probiotic bacteria, mainly LAB, are known to be able to increase IL-10 levels in the gut (Maldonado Galdeano et al., 2019). Interestingly, low transcriptional levels of IL-1β on the DSS groups treated with cheese (conventional or probiotic cheese) were observed. It is plausible to say that the downregulation of IL-1β can be associated with milk components in the cheese matrix, as noted by Kanwar et al. (2016). In addition, we can observe a systemic effect on the increase in IL-10 and decrease in IL-1β in the spleen of animals treated with probiotic cheese. In the local effects (lymph nodes), we see a decrease in IL-17 and an increase in IL-10, which corroborates with the low transcriptional colonic levels of IL-1β. This suggests that the effect of probiotic cheese may be associated with a decrease of pro-inflammatory Th1 and Th17 cytokines that can be linked to the enhanced production of IL-10 in the lymph nodes and spleen, as previously reported (Santos Rocha et al., 2014).

Foxp3^+^ Tregs are the subgroup of CD4^+^CD25^+^ T cells that have the capacity to inhibit the reactive effects of T cells by producing cytokine transforming growth factor β1 (TGF-β1) and IL-10 (Maldonado Galdeano et al., 2019). CD4^+^ T cells expressing FOXP3^+^, LAP^+^, and RORγt+ as analyzed in mouse spleens and lymph nodes (cecum and colon) show that treatment with probiotic cheese did not change regulatory T cell populations. Our work suggests that Foxp3^+^ Tregs are not responsible directly for the therapeutic effects of probiotic bacteria, despite increased levels of IL-10 (lymph nodes and spleen). It is plausible that the therapeutic effects of probiotic Minas Frescal cheese did not act *via* the adaptive immunity. However, IL-10 staining in regulatory T cells populations can be conducted to elucidate this hypothesis. Precisely, to confirm these results, it is necessary for other experiments to be able to indicate if the release of IL-10 is by innate immune cells such as macrophages and dendritic cells.

## Conclusion

We demonstrated that Minas Frescal cheese containing the well-characterized probiotic bacteria *L. lactis* NCDO 2118 was able to alleviate the severity of DSS-induced colitis in a mice model, limiting histopathological damages, restoring intestinal barrier by increased expression of gene related to tight junction protein, and modulating the cytokine production in mice. Probiotic Minas Frescal cheese was also able to prevent the degeneration of goblet cells and to reduce the inflammatory cell infiltration in the colon mucosa. Moreover, experimental probiotic cheese investigated in this work was able to produce high levels of bioactive peptides with antihypertensive, antioxidant, and antidiabetic activities. These results, together, open new perspectives for the development of probiotic functional foods for use in combination with conventional drugs or for use as an adjuvant therapy to enhance remission in UC patients.

## Data Availability Statement

The raw data supporting the conclusions of this article will be made available by the authors, without undue reservation.

## Ethics Statement

The animal study was reviewed and approved by Ethics Committee on Animal Experimentation of the Universidade Federal de Minas Gerais (CEUA-UFMG, Brazil, protocol 364/2018).

## Author Contributions

BC, VA, and FC conceived and designed the experiments. JA, LL, and AF performed and analyzed immunomodulatory experiments. MB was a major contributor to animal experimentation. EF performed, analyzed, and interpreted the histological analysis from colon slides. BC, GB, AG-G, and FC wrote the original draft. GJ and YL gave scientific advice. JG, RS, RR, MS, MF, EE, and AG-G manufactured the cheeses and performed centesimal and mineral composition. All authors contributed to data interpretation, drafted the manuscript, critically revised the manuscript, and approved its final version.

### Conflict of Interest

The authors declare that the research was conducted in the absence of any commercial or financial relationships that could be construed as a potential conflict of interest.
